# TMCO1 as an Endoplasmic Reticulum Calcium Load-Activated Channel: Mechanisms and Disease Implications

**DOI:** 10.3390/biom15081200

**Published:** 2025-08-20

**Authors:** Jingbo Wang, Panpan Zhu, Zhuohang Li, Xiaohui Su, Mingzhu Qi, Aimin Zhou, Xiangying Kong

**Affiliations:** 1 Institute of Chinese Materia Medica, China Academy of Chinese Medical Sciences, Beijing 100700, China; wangjingbo960911@163.com (J.W.); zhupanpan1998@163.com (P.Z.); lzhxx0224@163.com (Z.L.); xhsu@icmm.ac.cn (X.S.); 15077868613@163.com (M.Q.); 2 Department of Chemistry, Center for Gene Regulation in Health and Diseases, Cleveland State University, Cleveland, OH 44115, USA; a.zhou@csuohio.edu

**Keywords:** TMCO1, calcium, endoplasmic reticulum, cerebro-facio-thoracic dysplasia, osteoporosis

## Abstract

Calcium ions (Ca^2+^) play a vital role in many biological processes. Transmembrane and coiled-coil domain 1 (TMCO1) has been characterized as an endoplasmic reticulum (ER) transmembrane protein in recent years. It keeps the cytoplasm and ER’s Ca^2+^ homeostasis stable by acting as a novel calcium channel. Studies from different laboratories have revealed that the mutation or deficiency of TMCO1 is closely correlated with several diseases, including cerebro-facio-thoracic dysplasia (CFTD), glaucoma, premature ovarian failure (POF), osteoporosis, and cancer. Here, we review the characteristics of TMCO1 and its involvement in related diseases, which may provide useful information for developing therapeutic strategies for these diseases, as well as promote further research on this protein.

## 1. Introduction

Free calcium ions (Ca^2+^) are among the most prevalent intracellular signaling species, and their intracellular concentration must be precisely regulated to ensure the precision and fidelity of signal transmission [1]. The endoplasmic reticulum (ER) serves as a key regulatory hub for cellular Ca^2+^ signaling. Elevated Ca^2+^ levels promote the optimal activation of key enzymes regulated by ER calcium signaling, including calcineurin and calcium/calmodulin-dependent protein kinase II (CaMKII), among others [2,3,4]. Additionally, the ER dynamically modulates Ca^2+^ release and recovery through Ca^2+^ channels and calcium pumps to maintain intracellular Ca^2+^ homeostasis [5]. However, when the ER calcium release channels are inhibited or the calcium pump activity is upregulated, causing ER calcium overload [6,7,8], this disrupts the protein folding environment of the ER, resulting in the accumulation of unfolded or misfolded proteins and thereby inducing endoplasmic reticulum stress (ERS) [9]. ERS induces cell apoptosis by upregulating pro-apoptotic signals such as C/EBP homologous protein (CHOP) and cysteine–aspartic acid protease-3 (Caspase-3) [10,11,12]. Moreover, the precise modulation of ER calcium signaling is crucial for cellular physiological processes, including mitochondrial Ca^2+^ uptake, metabolic regulation, and the maintenance of redox balance [13]. Thus, the maintenance of ER and cytosolic Ca^2+^ homeostasis is essential for precise calcium signaling and normal cellular function [14].

**Figure 1:**
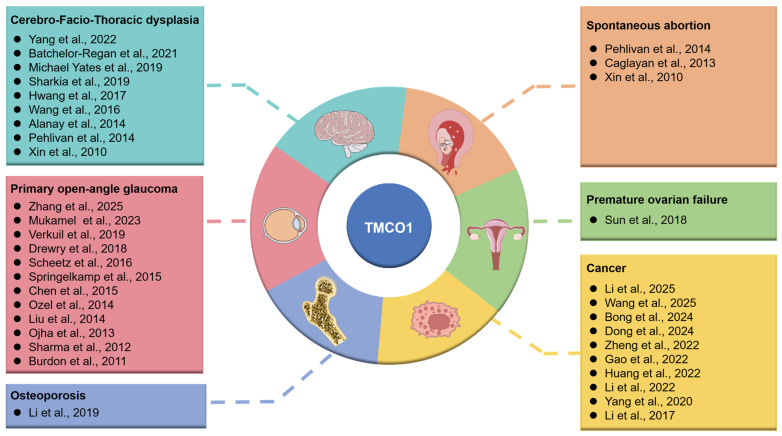
Current research status of TMCO1 in diseases. For detailed information on the literature referenced in the figure, please refer to the references section of this article [15,16,17,18,20,21,22,23,24,25,26,27,28,29,30,31,32,33,34,35,36,37,38,39,40,41,42,43,44,45,46,47,48,49].

Transmembrane and coiled-coil domain 1 (TMCO1) is a representative member of the domain of unknown function 841 (DUF841) superfamily, which is characterized by highly conserved amino acid sequences and widespread expression across all tissues from fetal to adult stages [15]. In recent years, with the widespread application of techniques such as cell biology, electrophysiology, and gene knockout, researchers have discovered that TMCO1 is an ER transmembrane protein that can sense Ca^2+^ concentrations within the ER [16]. TMCO1 oligomerizes into tetramers through homotetramerization in response to elevated calcium levels, facilitating calcium release to mitigate ER calcium overload and maintain intracellular calcium homeostasis [16]. This regulatory mechanism relies on the transmembrane domain of TMCO1, whose conformational changes facilitate the passive diffusion/release of Ca^2+^, thereby precisely modulating ER calcium levels. Indeed, mutations or deletions in the *TMCO1* gene can result in TMCO1 deficiency syndrome, a clinical phenotype highly consistent among multiple patients, characterized by developmental delay, typical craniofacial deformities, and skeletal abnormalities. Although mutations resulting in premature termination codons are found in all reported TMCO1-deficient syndrome patients, the severity and specific manifestations of the phenotype may vary depending on the specific location of the mutation, the efficiency of mRNA degradation, and the potential function of the truncated TMCO1 protein [15]. Moreover, with continuous research on the *TMCO1* gene, it has been found that functional loss mutations in the *TMCO1* gene are a key cause of cerebral facial thoracic dysplasia (CFTD). Meanwhile, a large number of genetic association studies have revealed that specific single-nucleotide polymorphisms (SNPs) in the *TMCO1* gene are significantly associated with the risk of developing primary open-angle glaucoma (POAG). Regulating *TMCO1* gene expression levels or gene knockout may lead to the development of osteoporosis, cancer, and premature ovarian failure (POF). Although current research suggests an association between TMCO1 functional loss mutations and spontaneous abortion, further studies are required [17,18,19,20] (Figure 1). This review summarizes the latest advances in our understanding of the structure and function of TMCO1, and its role in disease, with the aim of identifying new research directions and therapeutic opportunities.

## 2. The Protein Structure Characteristics of TMCO1

The amino acid sequence of TMCO1 is highly conserved across species [19]. Porcine and human TMCO1 share a closer genetic relationship, as indicated by phylogenetic analysis [50]. Although differences exist in *TMCO1* gene expression between humans and pigs, potentially due to species-specific biological activities in tissues, these differences suggest that pigs could serve as a viable animal model to explore the role of TMCO1 in humans. Furthermore, *Tmco1* knockout mice display key clinical features similar to human disorders linked to mutations in this gene, making them ideal models for investigating its biological functions [41].

### 2.1. Two Different Subtypes of TMCO1

*TMCO1* mRNA harbors two translation initiation sites, resulting in two protein isoforms: one with 188 amino acids and the other with 239 amino acids [51]. TMCO1/188 consists of two transmembrane domains, whereas TMCO1/239 comprises three transmembrane domains, attributed to an additional 51 amino acids at the N-terminus. This structural variation may confer distinct functional properties to the two isoforms. TMCO1/188 can form Ca^2+^ channels via tetramerization to regulate ER calcium levels, while the function of TMCO1/239 remains largely unknown. Studies have indicated that *TMCO1* mRNA primarily initiates translation from downstream start codons, predominantly producing TMCO1/188, potentially linked to the cellular demand for calcium homeostasis. In different cell types or tissues, TMCO1/239 may have a more significant role, with its expression potentially linked to specific physiological processes or developmental stages [51]. Furthermore, frameshift mutations in the *TMCO1* gene can lead to syndromes such as craniofacial malformations, skeletal abnormalities, and developmental delays. This may be related to the roles of Ca^2+^ in cell signaling, cell differentiation, and embryonic development. Consequently, we hypothesize that functional abnormalities of TMCO1/188 may lead to Ca^2+^ homeostasis imbalance, which in turn disrupts embryonic development and organ formation. As for TMCO1/239, although its precise function remains unclear, its unique structural features, including the additional 51 amino acids and three transmembrane domains, suggest that it may either complement or function independently of TMCO1/188 in specific physiological processes.

### 2.2. TMCO1 Belongs to the YidC/Alb3/Oxa1 Family

The YidC/Alb3/Oxa1 family has been expanded through the study of the structural domain of DUF106. As a result of the presence of DUF106, TMCO1 has been classified as a newly identified component of the YidC/Alb3/Oxa1 family in the ER. Proteins in the Oxa1 superfamily display similar membrane topologies and core structural characteristics [52]. Topology mapping and coevolution-based modeling have demonstrated that TMCO1 is a highly conserved protein, structurally akin to Oxa1, comprising a core region with three transmembrane domains, a coiled helix facing the cytoplasm, and a C-terminus. These features closely resemble those of other members of the YidC/Alb3/Oxa1 family, suggesting that TMCO1 shares a common ancestor with this family and has retained key structural domains throughout evolution.

Biochemical analysis of human TMCO1 revealed that TMCO1 interacts with the Sec61 translocon and ribosomes [53]. Through immunoprecipitation experiments, researchers successfully isolated the TMCO1–ribosome complex from human HEK293 cells, confirming the binding of TMCO1 to both ribosomes and the Sec61 translocon. Additionally, cryo-EM technology revealed a ribosome-associated complex of approximately 360 kDa, which includes Sec61, TMCO1, CCDC47, Nicalin, and TMEM147 [53]. In vitro studies have demonstrated that TMCO1 exhibits an intrinsic affinity for ribosomes, similar to other members of the YidC/Alb3/Oxa1 family [54]. According to proteomics research, it is speculated that cytoplasmic kinases can phosphorylate serine residues in the coiled coil and C-terminal domain of TMCO1 [55]. Furthermore, the function of the TMCO1 protein is similar to that of YidC in bacteria, potentially participating in co-translational insertion, folding, and assembly of membrane proteins [54]. However, although these findings reveal the potential role of TMCO1 in membrane protein biosynthesis, its specific function requires further investigation. For example, the calcium leakage activity of TMCO1 and its association with protein synthesis remain unclear. TMCO1 has been found to regulate Ca^2+^ concentration in the ER, but its specific mechanism of action in membrane protein biosynthesis has not yet been fully elucidated. Additionally, although TMCO1 shares similar intrinsic affinities with other members of the YidC/Alb3/Oxa1 family, its specific role in the cotranslational process requires further investigation. Future studies should further explore whether TMCO1’s calcium leakage activity influences the folding and insertion of membrane proteins by regulating Ca^2+^ concentrations in the ER, as well as how TMCO1 collaborates with Sec61 and other cofactors to promote the biosynthesis of transmembrane proteins.

## 3. TMCO1 Is a Ca^2+^ Channel Activated by Ca^2+^ Overloading in the ER

Ca^2+^ functions as a second messenger, participating in signaling pathways that regulate various cellular processes [56,57]. Calcium homeostasis is maintained not only through the coordinated transport of calcium channels, calcium pumps, and ion exchangers, but also through buffering by calcium-binding proteins and the storage and release of calcium by organelles such as the ER and mitochondria, which together regulate the balance of intracellular Ca^2+^ concentration and maintain normal cellular function [58,59].

### 3.1. TMCO1-Mediated ER Ca^2+^ Efflux

**Figure 2:**
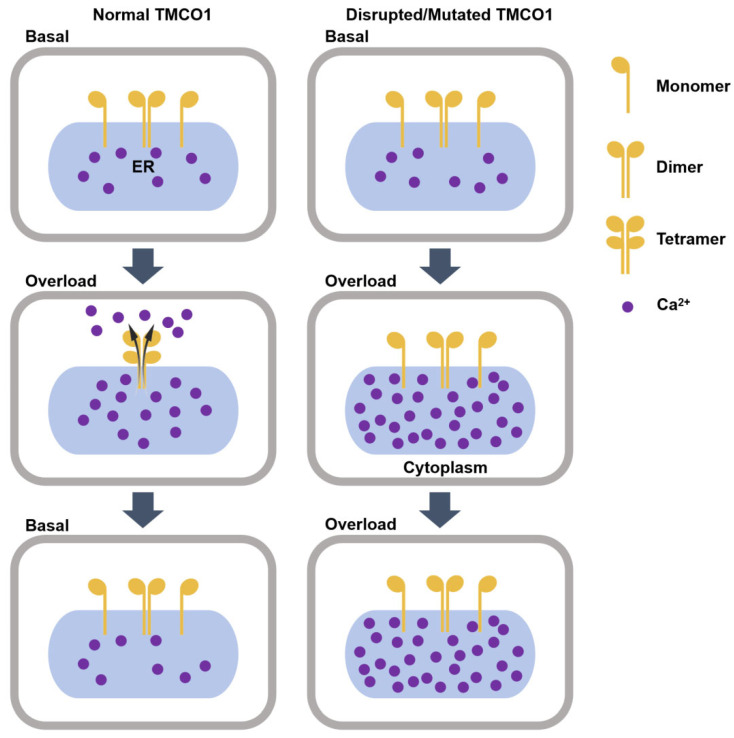
The molecular mechanism of the TMCO1 function as an ER Ca^2+^ load-activated Ca^2+^ channel. In response to Ca^2+^ overloading in the ER, TMCO1 homotetramerizes and forms the Ca^2+^-selective ion channel on the ER, and subsequently releases Ca^2+^. After the Ca^2+^ concentration in the ER returns to normal, the tetramers disaggregate, and the Ca^2+^ channel activity disappears. Disrupted or mutated TMCO1 does not respond to Ca^2+^ overload in the ER, resulting in cellular dysfunction.

In 2016, *Cell* published a seminal study that uncovered the pivotal role of TMCO1, a novel Ca^2+^ channel protein, in the ER. The study suggested that TMCO1’s primary function is to prevent the excessive accumulation of Ca^2+^ in the ER, thereby maintaining intracellular calcium homeostasis [16]. The molecular mechanism underlying TMCO1 function is as follows: it senses elevated calcium levels in the ER, forms a tetramer with Ca^2+^ channel activity, and mediates the efflux of Ca^2+^ from the ER through the formation of calcium-selective channels. As the Ca^2+^ levels in the ER gradually normalize, the tetramer dissociates, leading to the cessation of Ca^2+^ channel activity [18]. In contrast, when the TMCO1 function is disrupted/mutated, it cannot form tetramers with Ca^2+^ channel activity, resulting in Ca^2+^ overload in the ER [16]. Thus, cells maintain calcium homeostasis by regulating the oligomerization and disassembly of TMCO1 protein tetramers (Figure 2).

The inositol 1,4,5-triphosphate receptor (IP3R) is another important Ca^2+^ release channel in the ER. In contrast to TMCO1, its primary role is to amplify and transmit intracellular Ca^2+^ signals [60]. IP3R activation is dependent on inositol 1,4,5-triphosphate (IP3) in the cytoplasm. When extracellular signals (e.g., hormones or neurotransmitters) activate receptors on the cell membrane, IP3 is generated via signal transduction, which then binds to IP3R to activate the channel and trigger Ca^2+^ release from the ER into the cytoplasm. It is important to note that TMCO1 may indirectly affect the calcium release induced by IP3 through the maintenance of ER calcium homeostasis. Atakpa et al. [61] demonstrated that when the expression level of TMCO1 was reduced by 67% using CRISPR/Cas9 technology, calcium release induced by carbachol (which is known to primarily act through the IP3 receptor pathway) increased. This phenomenon may result from TMCO1 deficiency disrupting ER calcium homeostasis, which could lead to ER calcium overload and subsequently enhance IP3R sensitivity or increase its open probability. However, whether TMCO1 directly modulates IP3R function requires further validation.

### 3.2. Effects of TMCO1 Deficiency on Cellular Functions

ER calcium overload exerts significant impacts on cellular functions. Moderate calcium overload can activate intracellular signaling pathways, participating in cellular stress responses and adaptive regulation [5]. However, excessive calcium overload may induce ERS, leading to cellular dysfunction or even apoptosis [62]. Under physiological conditions, ER Ca^2+^ is imported from the cytosol into the ER lumen via the SERCA pump, while IP3Rs and ryanodine receptors (RYRs) mediate Ca^2+^ release from the ER. Additionally, passive leak channels on the ER membrane contribute to maintaining calcium homeostasis [7]. Disruption of these regulatory mechanisms may cause a pathological elevation of ER calcium concentration, resulting in ER calcium overload.

**Figure 3:**
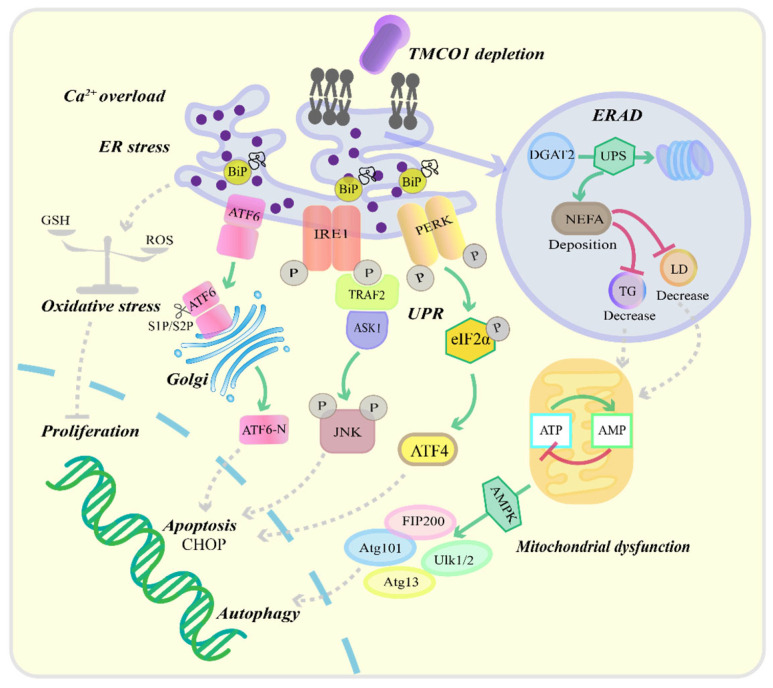
Effect of TMCO1 deletion on cell function. Depletion of TMCO1 leads to an overload of ER Ca^2+^, which subsequently induces ERS. ERS promotes the dissociation of BiP from IRE1α, PERK, and ATF6, thereby initiating a cascade activation of the UPR signaling pathway. Upon BiP dissociation from IRE1α, IRE1α undergoes homodimerization and autophosphorylation. The activated IRE1α then recruits TRAF2, thereby activating the ASK1-JNK signaling cascade and ultimately inducing cell apoptosis. The dissociation of BiP from PERK leads to PERK activation and subsequent phosphorylation of eIF2α, which enhances the translation of the transcription factor ATF4, upregulates the expression of the pro-apoptotic factor CHOP, and triggers cell apoptosis. ERS can induce the translocation of ATF6 to the Golgi apparatus, where it is cleaved by S1P and S2P, resulting in the release of ATF6-N into the nucleus, which promotes the expression of pro-apoptotic genes, such as CHOP, thereby exacerbating cell apoptosis. Absence of TMCO1 also triggers the ERAD pathway. The degradation of DGAT2 via ERAD results in a reduction of liver LD and TG levels, alongside the accumulation of NEFA. These changes lead to a reduction in mitochondrial ATP synthesis and the activation of AMPK, which subsequently induces Atg1-mediated autophagy (Ulk1/Ulk2). Furthermore, the absence of TMCO1 can disrupt redox balance, increase mitochondrial ROS production, and ultimately impair cellular function. →—increase or activation, ┤—decrease or inhibition.

TMCO1 is an ER Ca^2+^ load-activated Ca^2+^ channel that plays a pivotal role in maintaining ER calcium homeostasis [16] (Figure 3). Wang et al. [16] demonstrated that ethanol-induced ER calcium overload triggers TMCO1 tetramerization, forming functional calcium release channels. TMCO1 deficiency impairs Ca^2+^ efflux from the ER, resulting in ER calcium overload and subsequent ERS [63]. This phenomenon may be attributed to elevated ER Ca^2+^ levels interfering with the molecular chaperone binding immunoglobulin protein (BiP), impairing its ability to bind and stabilize nascent polypeptide chains. Consequently, this disruption promotes the accumulation of misfolded proteins [64,65]. During ERS, key sensor proteins of the unfolded protein response (UPR)—including protein kinase RNA-like ER kinase (PERK), inositol-requiring enzyme 1 α (IRE1α), and activating transcription factor 6 (ATF6)—become activated [66]. Normally bound to the chaperone protein BiP, these sensors initiate UPR signaling upon BiP dissociation during stress [67]. In TMCO1-deficient cells, calcium overload promotes BiP dissociation from IRE1α, leading to IRE1α dimerization, autophosphorylation, and activation of the TRAF2-ASK1-JNK apoptotic pathway [68,69]. Similarly, BiP dissociation from PERK results in eIF2α phosphorylation, suppressing global protein synthesis while enhancing ATF4 translation. Prolonged stress induces ATF4-mediated CHOP expression, promoting apoptosis [67,70]. ATF6, a type II ER transmembrane protein, undergoes S1P/S2P-mediated cleavage during stress, releasing its transcriptionally active N-terminal domain (ATF6-N) that upregulates pro-apoptotic genes such as CHOP [67]. While direct evidence linking TMCO1 to PERK/ATF6 activation remains limited, we propose that TMCO1 deficiency-induced calcium overload may comprehensively activate UPR pathways, ultimately leading to apoptosis.

Additionally, TMCO1 deficiency activates endoplasmic reticulum-associated degradation (ERAD) through the ubiquitin–proteasome pathway. Experimental evidence demonstrates that TMCO1 depletion promotes ERAD-mediated degradation of diacylglycerol O-acyltransferase 2 (DGAT2), the rate-limiting enzyme in triglyceride (TG) biosynthesis. This molecular event consequently reduces hepatic lipid droplet (LD) formation and TG accumulation, while significantly increasing non-esterified fatty acid (NEFA) levels [63]. The pathological accumulation of NEFA impairs mitochondrial oxidative phosphorylation, leading to decreased ATP production and consequent elevation of the AMP/ATP ratio [71]. This energy stress activates AMP-activated protein kinase (AMPK), which initiates autophagy through phosphorylation of the Atg1 (Ulk1/Ulk2) complex [72,73]. Meanwhile, TMCO1 knockout induces oxidative stress by disrupting cellular redox homeostasis, resulting in excessive mitochondrial reactive oxygen species (ROS) generation [17]. These metabolic disturbances ultimately impair cellular proliferation, exacerbate inflammatory responses, and promote cell death pathways [74]. As an ER transmembrane protein, TMCO1 dysfunction has been pathogenically linked to multiple human disorders through these molecular mechanisms.

## 4. TMCO1 Mutation and Diseases

TMCO1 plays a critical role in regulating Ca^2+^ concentration within the ER. Mutations or disruptions of TMCO1 are associated with various diseases [16] (Table 1).

### 4.1. Cerebro-Facio-Thoracic Dysplasia

CFTD is an autosomal recessive genetic disorder characterized by cranial skeletal abnormalities and central nervous system developmental defects [22]. In 1975, Pascual-Castroviejo first reported three patients who exhibited prominent intellectual disability, typical craniofacial anomalies, and abnormalities of the spine and ribs. This condition was later defined as CFTD [75]. With further research on CFTD, its phenotype has been expanded to include hypoplasia of the corpus callosum and cerebellar vermis, cleft lip and palate, hypothyroidism, colobomas of the optic nerve, ptosis, and small conical teeth [76].

**Figure 4:**
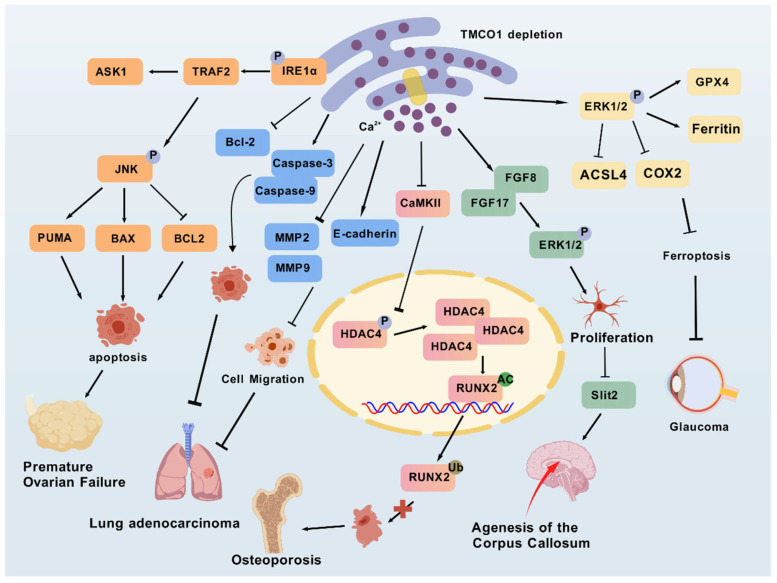
Role of TMCO1 in disease. The figure provides a summary of the molecular pathways implicated in TMCO1 depletion in various diseases, as discussed in this review, including the FGF/ERK pathway, the CaMKII-HDAC4-RUNX2 signaling axis, the IRE1α-TRAF2-ASK1-JNK signaling axis, and the ERK/GPX4/COX2 pathway. The graphics were created with BioGDP.com [78]. →—increase or activation, ┤—decrease or inhibition.

In 2010, Xin et al. [15] reported 11 patients with a TMCO1 homozygous frameshift mutation (C.139–140 delAG), which results in the deletion of two nucleotides (AG) at positions 139 and 140 in the coding region of TMCO1, causing a frameshift and introducing a premature stop codon at amino acid position 47 (p.Ser47Ter). The clinical manifestations include craniofacial dysmorphism, skeletal abnormalities, and intellectual disability, which the authors referred to as TMCO1 deficiency syndrome [15]. In recent years, with the application of whole-exome sequencing (WES) in human and clinical genetics research, Pehlivan et al. [27] demonstrated via WES that CFTD and TMCO1 deficiency syndrome exhibit highly overlapping phenotypes, including distinctive facial dysmorphism, central nervous system abnormalities, and thoracic skeletal malformations. In 2016, Wang et al. demonstrated the phenotypes in *Tmco1* knockout mice [16]. The resulting TMCO1 deficiency syndrome, caused by mutations in this gene, is characterized by craniofacial dysmorphism, skeletal dysplasia, intellectual disability, and ataxia. They proposed that these clinical manifestations are associated with disrupted Ca^2+^ homeostasis in the ER [15]. In fact, TMCO1 dysfunction not only leads to craniofacial anomalies, spinal deformities, and cognitive impairment, but also causes various other clinical manifestations resembling CFTD, including epilepsy and gingival hyperplasia [24,25,26,39]. Caglayan et al. reported agenesis of the corpus callosum and cerebellar herniation in Turkish patients with TMCO1 deficiency syndrome, which are also among the most common features observed in CFTD patients [40]. In the study by Yang et al. [21], the authors elucidated the mechanism behind the underdevelopment of the corpus callosum in TMCO1 deficiency. This is because Ca^2+^ overload in the ER impairs the normal function of calcium release-activated calcium channels (SOCE), leading to abnormal elevation of intracellular Ca^2+^ signaling. This subsequently activates CaMKs/CREB pathways, causing significant upregulation of FGF8 and FGF17 transcription and protein expression, ultimately resulting in hyperphosphorylation of ERK1/2. This cascade promotes aberrant glial cell migration and excessive midline gray matter accumulation, which secretes Slit2 to inhibit axonal growth, thereby causing corpus callosum agenesis (Figure 4). Furthermore, patients homozygous for the TMCO1 pathogenic mutation c.292_293del (p.Ser98*) exhibit the CFTD phenotype [23]. Previously, this mutation had been reported solely in isolated Amish populations. However, a recent study identified two siblings who developed the clinical symptoms of CFTD due to a unique homozygous pathogenic variation in the *TMCO1* gene, p.Arg114* [76]. Although TMCO1 deficiency syndrome was once considered a distinct disorder, increasing evidence suggests that it may be classified within the gene-heterogeneous CFTD dysplasia spectrum [26]. While TMCO1 deficiency syndrome and CFTD share similar clinical symptoms, they are distinct conditions. TMCO1 deficiency syndrome is considered a subtype of CFTD, and some CFTD patients may harbor additional genetic mutations. For instance, recent MITOMICS data identified a homozygous stop codon variant (c.75G > A, p.Trp25 *) in the RAB5IF gene, a novel mutation associated with the CFTD phenotype through modulation of TMCO1 protein expression [77].

Overall, these findings underscore the critical role of TMCO1 in neural and skeletal development, highlighting its function as a key gene in the genetically heterogeneous spectrum of CFTD. In addition, the loss of TMCO1 function results in various pathological manifestations due to the critical role of TMCO1 in intracellular calcium homeostasis, the differential sensitivity of different tissues to abnormal calcium homeostasis, the heterogeneity of gene mutations, and the cell type and tissue specificity. Notably, the heterogeneity of clinical manifestations in TMCO1 deficiency is not solely attributable to TMCO1 mosaic mutations. Even without mosaic mutations, variations in TMCO1 expression levels across tissues, the complexity of calcium signaling pathway interactions, and potential compensatory mechanisms in specific tissues could contribute to the diverse clinical manifestations among individuals.

### 4.2. Glaucoma

Glaucoma is a neurodegenerative disease characterized by the progressive loss of retinal ganglion cells, causing distinctive optic nerve head cupping and peripheral visual impairment [79]. Among them, POAG is the most prevalent and has become the leading cause of irreversible blindness worldwide [80]. Studies have shown that the *TMCO1* gene is significantly expressed in retinal tissues, trabecular network structures, and human ciliary bodies [29]. Molecular genetic studies indicate that individuals with the TMCO1 rs4656461-G risk allele have an increased likelihood of developing POAG compared to those without the allele. This may be associated with the downregulation of *TMCO1* gene expression or protein function by TMCO1 rs4656461-G, which in turn disrupts intracellular calcium homeostasis [31].

Genome-wide association studies (GWAS) have identified over 100 variants associated with POAG, which are widely distributed across multiple distinct genes or genomic regions [81,82]. Notably, common variants within the *TMCO1* gene region have been linked to elevated intraocular pressure (IOP) levels [36]. Studies have confirmed that IOP, as a critical factor in POAG, exhibits high heritability, making it a primary target for POAG treatment [83,84]. Thus, TMCO1 may contribute to POAG pathogenesis by modulating IOP levels [85]. A GWAS and IOP meta-analysis involving over 6000 individuals of European ancestry further validated the strong association between TMCO1 and IOP [34]. Recent research has revealed that extracellular matrix (ECM) deposition is a key factor in increasing trabecular meshwork resistance, impairing aqueous humor outflow, and elevating IOP [20]. Upon TMCO1 knockout, ER Ca^2+^ homeostasis is disrupted, leading to altered Ca^2+^ distribution. This change activates Ca^2+^-dependent signaling pathways, promoting ERK1/2 phosphorylation, which upregulates the expression of the antioxidant enzyme GPX4 and the iron storage protein ferritin, thereby enhancing cellular antioxidant capacity and reducing the accumulation of lipid peroxides and free iron ions. Concurrently, ERK1/2 activation downregulates the expression of the lipid metabolism enzyme ACSL4 and the inflammation-related enzyme COX2, mitigating lipid peroxide accumulation and suppressing inflammatory responses, ultimately inhibiting ferroptosis [20]. This process not only reduces ECM deposition but also decreases trabecular meshwork resistance, effectively lowering IOP and delaying POAG progression. It is important to note that ERK1/2 plays a multifaceted role in POAG, as it also promotes retinal ganglion cell survival [86]. Moreover, genetic analysis of risk alleles associated with POAG has identified TMCO1 SNPs in Korean patient cohorts as representative of POAG-associated mutations [87]. Further investigation revealed that TMCO1 SNPs have been found to be significantly associated with POAG, even after adjustments for age, gender, and Bonferroni correction [88]. A study on the genetic variation of the *TMCO1* gene and POAG also confirmed that rs4656461 (G > A substitution downstream of TMCO1) on chromosome 1q24 near the *TMCO1* gene is highly correlated with POAG risk [37]. A meta-analysis showed that rs7555523 (C > A upstream of TMCO1) on chromosome 1q24.1 is significantly associated with IOP [89]. Additionally, in severe POAG patients in Australia, rs4656461 and rs7555523 were found to coexist in some cases, with a distance of 31,774 base pairs between them [32]. In neural network analysis, rs4656461 was identified as the most significant genetic variant in the development of POAG, as patients with POAG-like symptoms carried the rs4656461 risk allele [88]. Another study reported that individuals carrying the rs4656461 risk allele in the *TMCO1* gene are typically diagnosed with POAG at a young age [37]. Additionally, both Kaplan–Meier analysis and the Cox proportional hazards model indicate that the TMCO1 genotype is clinically significant for assessing glaucoma risk [31].

In summary, these results suggest that the *TMCO1* gene may serve as a genetic marker for susceptibility to POAG. Current research suggests that mutations in the *TMCO1* gene are associated with both CFTD and POAG. This suggests that dysfunction of the *TMCO1* gene may play an important role in both diseases. However, whether CFTD is associated with POAG has not yet been reported. This observation may reflect limitations in clinical screening or the heterogeneity of pathogenic mechanisms underlying TMCO1 mutations. Prospective ophthalmic studies in CFTD cohorts may help to clarify this issue in the future.

### 4.3. Osteoporosis

Osteoporosis is a systemic skeletal disorder characterized by reduced bone mass and compromised tissue structure, which increases bone fragility and fracture risk [90]. It is a complex disease influenced by both genetic and environmental factors. Osteoporosis affects individuals of all genders and ethnicities, with postmenopausal women being at the highest risk [91]. Disruption of Ca^2+^ homeostasis in the ER is associated with several severe bone disorders [92,93]. Studies have shown a significant reduction in TMCO1 expression in bone samples from both osteoporotic patients and animal models. This downregulation is correlated with impaired osteoblast function [39]. Osteoblastic differentiation and bone formation are inhibited in the absence of TMCO1 expression [39].

As a key mediator of Ca^2+^ signaling, CaMKII regulates multiple stages of the calcium cycle, thus influencing various cellular functions, including proliferation and migration [94]. Studies have shown that calcium release mediated by TMCO1 activates the Ca^2+^/CaMKII signaling pathway, subsequently promoting bone formation in osteoblasts [39]. Furthermore, suppression of TMCO1 expression in A549 cells leads to Ca^2+^ overload, subsequently downregulating CaMKII expression [48]. Runx2 is a critical transcription factor required for osteoblast function, while HDAC4 inhibits the stability and transcriptional activity of Runx2 via deacetylation, thereby affecting osteogenesis [39]. Studies have demonstrated that TMCO1 deficiency in osteoblasts disrupts Ca^2+^ homeostasis in the ER, promoting CaMKII-HDAC4-induced degradation of Runx2, a process dependent on local Ca^2+^ signaling [39]. Taken together, these findings suggest that TMCO1 is crucial for bone formation. Further investigations are needed to explore the potential of TMCO1 as a diagnostic marker or therapeutic target for osteoporosis.

### 4.4. Cancer

It has been shown that the biological activity of cancer cells, particularly their ability to proliferate and migrate, is significantly affected by the disruption of intracellular Ca^2+^ homeostasis [58]. Furthermore, accumulating evidence indicates that Ca^2+^-mediated signaling pathways are strongly linked to cancer development [95]. TMCO1, as an ER calcium channel protein, regulates the function of p53 apoptosis-stimulating protein inhibitor (iASPP) by competing with the ER-associated E3 ligase Gp78. Inhibition of the iASPP-TMCO1 axis can induce the release of cytoplasmic Ca^2+^, thereby promoting cell apoptosis and inhibiting tumor growth [44]. These findings suggest that TMCO1 may play a critical role in cancer biology.

Indeed, TMCO1 is highly expressed in most carcinomas. Research has shown that TMCO1-AS1, a newly identified lncRNA with aberrant expression in hepatocellular carcinoma, correlates positively with α-fetoprotein (AFP) levels, tumor stage, and cellular differentiation grade, acting as a predictive biomarker for both overall survival (OS) and recurrence-free survival (RFS) [46]. In a study of head and neck squamous cell carcinoma (HNSCC), Li et al. [47] suggested that TMCO1 acts as a tumor antigen associated with prognosis and the infiltration of antigen-presenting cells in HNSCC. They also highlighted its potential immunostimulatory properties, which could be harnessed by antigen-presenting cells to trigger anti-tumor immune responses. Gao et al. [45] observed increased levels of *TMCO1* mRNA and protein in glioblastoma multiforme tissue. They found that TMCO1 knockout can induce apoptosis and inhibit cell proliferation in U87 and U251 cells, thereby suppressing glioma malignancy and prolonging survival in glioma patients. Bong et al. [18] found that TMCO1 is highly expressed in breast cancer, where its expression is associated with poor prognosis in basal-type breast cancer. The study by Yang et al. [48] revealed that TMCO1 deficiency leads to intracellular Ca^2+^ overload, which subsequently promotes apoptosis in lung adenocarcinoma cells by downregulating Bcl-2 expression and upregulating the levels of Caspase-3 and Caspase-9. Concurrently, TMCO1 depletion was found to reduce the expression of MMP-2 and MMP-9 while increasing E-cadherin levels, thereby inhibiting the epithelial–mesenchymal transition (EMT) process and ultimately suppressing cell migration capacity. These findings suggest that targeting TMCO1 deficiency may represent a potential therapeutic strategy for the treatment of lung adenocarcinoma. Furthermore, TMCO1 is also a novel tumor suppressor. In 2007, Li et al. [49] discovered that TMCO1 can recruit PH domain leucine-rich repeat-containing protein phosphatase 2 (PHLPP2), leading it to bind to pAKT1 (S473) and dephosphorylate it, thereby inhibiting the AKT signaling pathway and suppressing the growth of invasive urothelial bladder carcinoma (UBUC).

In summary, these studies suggest that TMCO1 may play either a pro-cancer or anti-cancer role in various cancers, influencing cancer cell proliferation, apoptosis, and immune response.

### 4.5. Premature Ovarian Failure

POF is a heterogeneous disorder characterized by the loss of normal ovarian function before the age of 40, marked by elevated gonadotropin levels, decreased estradiol levels, and impaired ovarian reserve function [96]. Granulosa cells (GCs) within ovarian follicles play essential roles in folliculogenesis, providing both nutritional support and steroid hormone production [97]. Recent studies have identified TMCO1 as a critical regulator of follicular development. This protein maintains normal folliculogenesis by preserving Ca^2+^ homeostasis in the ER of GCs [41]. Animal studies have demonstrated that TMCO1-knockout mice exhibit significantly increased GC apoptosis, leading to insufficient follicular nourishment and subsequent follicular atresia [41]. Further investigations by Sun et al. revealed that female mice with TMCO1 deficiency display progressive follicular depletion, arrested follicular development, and reduced fertility—phenotypes that closely resemble the clinical manifestations of human POF [41]. At the molecular level, TMCO1 deficiency triggers calcium overload in the ER of GCs, which subsequently activates the IRE1α-dependent UPR. The aberrant activation of this signaling pathway exacerbates cellular apoptosis while simultaneously elevating reactive oxygen species (ROS) levels in both mitochondria and cytoplasm, ultimately promoting follicular atresia and ovarian dysfunction [41]. These findings not only elucidate the pivotal regulatory role of TMCO1 in follicular development but also provide novel mechanistic insights into the pathogenesis of POF.

Currently, there is no information linking POF to TMCO1 mutations in databases such as the GWAS Catalog. However, variants in the *TMCO1* gene have emerged as a primary factor for POF in humans, as inferred from animal studies. Although mice share a large number of genes and physiological mechanisms with humans, they have significant differences in genetic background, metabolic pathways, natural disease progression, and drug response, which may lead to biases in inferring human diagnosis from mouse experiments. Therefore, it remains essential to examine TMCO1 across diverse POF patient cohorts to identify pathogenic mutations and advance diagnostic and prognostic strategies for POF.

### 4.6. Spontaneous Abortion

Spontaneous abortion refers to the involuntary termination of pregnancy that occurs before 20 weeks of gestation. It is a common complication in early pregnancy, occurring in approximately 10–15% of clinically diagnosed pregnancies [98]. It causes significant physical and psychological trauma to women, and the risk factors that can be effectively addressed remain limited.

ERS and cell apoptosis may be crucial factors in regulating miscarriage. Studies have shown [99] that in *Eif2s1*^tm1RjK^ mice, ERS leads to a significant reduction in the number of trophoblast cells at the blastocyst stage, resulting in developmental delay. The sustained activation of ERS and the UPR pathway drives the early differentiation of trophoblast stem cells, leading to reduced volume and inadequate vascularization of the placental labyrinth, which in turn results in embryonic growth restriction or mid-pregnancy loss. Wan et al. [100] found that activation of the Caspase-2/Caspase-3-dependent mitochondrial apoptosis pathway induces excessive apoptosis of placental trophoblast cells, resulting in miscarriage.

Furthermore, genetic abnormalities may also contribute to miscarriage. Xin et al. [15] reported a 22% incidence of first-trimester spontaneous abortion in fetuses with TMCO1 deficiency syndrome among affected families. This finding was derived from a comprehensive investigation of 11 families with TMCO1-deficient individuals, encompassing a total of 45 pregnancies, among which 10 cases of first-trimester spontaneous abortion were documented. Although the precise role of TMCO1 in embryonic development and gestation remains undefined, its high conservation and ubiquitous expression across nearly all human embryonic and adult tissues suggest that TMCO1 may be involved in developmental regulation. Disruption of TMCO1 may contribute to spontaneous abortion, particularly in families affected by either TMCO1 defect syndrome or CFTD [27].

In summary, these findings open new avenues for exploring the molecular mechanisms underlying early spontaneous abortion and suggest the necessity of targeted genetic screening for individuals at high risk of pregnancy complications or with a family history of TMCO1-related syndromes.

## 5. Conclusions and Perspectives

Taken together, TMCO1, an ER transmembrane protein, plays a crucial role in maintaining ER Ca^2+^ homeostasis. Numerous studies have confirmed that mutations or functional deficiencies in the *TMCO1* gene, as well as abnormal downregulation of its protein expression, can lead to the occurrence of diseases related to calcium homeostasis disorders. In addition to the well-known associations with CFTD, glaucoma, and POF, recent studies have highlighted the involvement of TMCO1 in the development of osteoporosis and various cancers. These findings suggest that TMCO1 may serve as a promising biomarker and therapeutic target for these diseases.

Although current research has revealed the important role of TMCO1 in calcium-associated diseases, its potential as a therapeutic target requires further exploration. It is urgent to elucidate the molecular mechanisms by which TMCO1 mutations lead to disease phenotypes through functional studies, particularly by establishing conditional gene knockout animal models and cell-specific knockout studies to validate their pathological effects. Additionally, while TMCO1 is localized to the ER, its biological functions in other organelles, such as mitochondria, remain unclear. Further investigations employing high-resolution imaging and multi-omics technologies are required to precisely elucidate the roles of TMCO1 in various tissues and organelles. Moreover, drug development targeting TMCO1 remains in its early stages. We suggest integrating high-throughput small molecule screening with organoid disease models to assess its therapeutic potential, particularly in bone metabolism and cancer. These systematic investigations will provide crucial evidence for elucidating the multifaceted biological functions of TMCO1 and for developing targeted therapeutic strategies.

## Figures and Tables

**Table 1:** Summary of the TMCO1 mutation-associated diseases.

Diseases	Cells/Tissues	Mutation/Disruption	Regulation of Proteins/Pathways	Effects	Symptom	Ref.
Cerebro-facio-thoracic dysplasia	adult and fetal tissues of humans	TMCO1 (C.139–140 delAG)	Premature cessation of Ser47 of the protein	A highly truncated protein (p.Ser47Ter), only a quarter of its original length	Craniofacial deformity, spinal abnormalities, mental retardation, epilepsy, hyper-gingival growth, etc.	[16,25,39]
Primary open-angle glaucoma	Ocular tissues	rs4656461/rs7555523 TMCO1 risk allele	MYOC expression	Intraocular pressure (IOP) changes	Characteristic bending of the optic nerve head concurrent with diminished peripheral visual perception	[20,33]
Osteoporosis	Osteoblast	TMCO1 deletion/loss-of-function mutation or downregulation	Decreasing the CAMKII expression level and promoting CaMKII-HDAC4-mediated RUNX2 degradation	Inhibition of osteoblastic differentiation and bone formation	Decreasing bone mass and degrading microstructure of the bone tissue	[39,48]
Cancer	Cancer cells	*TMCO1* gene overexpression	Calcium signaling regulation/Inhibiting the AKT signaling pathway	Promotion/inhibition of cancer cell growth and migration	Mass effects, organ obstruction/destruction, invasive growth, dissemination, overcrowding of normal tissue, metastasis formation, pain, metabolic effects (cachexia), paraneoplastic syndromes, etc .	[17,43]
Premature ovarian failure	Granulosa cells (GCs)	TMCO1 deletion or loss-of-function mutation	As a Ca^2+^ channel triggered by Ca^2+^ loads	Increased amounts of ROS and apoptosis caused by ERS	Amenorrhea before age 40 with elevated gonadotropin and luteinizing hormone levels	[41]
Spontaneous abortion	Human adult and fetal tissues	TMCO1 functional defect	Unclear	Unclear	Termination of pregnancy for less than 28 weeks of pregnancy and less than 1000 g of fetal weight	[15,27]

## Data Availability

No new data were created or analyzed in this study. Data sharing is not applicable to this article.
